# Effects of extraction methods for a new source of biostimulant from *Sargassum horneri* on the growth of economically important red algae, *Neopyropia yezoensis*

**DOI:** 10.1038/s41598-022-16197-0

**Published:** 2022-07-13

**Authors:** Sol Han, Ji-Sook Park, Schery Umanzor, Charles Yarish, Jang K. Kim

**Affiliations:** 1 Department of Marine Science, Incheon National University, Incheon, 22012 Korea; 2 Research Institute of Basic Sciences, Incheon National University, Incheon, 22012 Korea; 3 College of Fisheries and Ocean Sciences, University of Alaska Fairbanks, Juneau, AK 99801 USA; 4 Department of Ecology and Evolutionary Biology, University of Connecticut, Stamford, CT 06901-2315 USA

**Keywords:** Abiotic, Marine biology

## Abstract

*Sargassum horneri* is a major bloom forming species in Korea and China. It is important to find a way to utilize the huge biomass of *Sargassum horneri* in the region. Seaweed-derived biostimulants are primarily derived from brown algae and are known to improve terrestrial crop growth and tolerance to abiotic stresses. *Neopyropia yezoensis* is the most important seaweed cultured species in Korea, and research is required to increase heat resistance as a solution against climate change. In this study, various extraction methods were used to obtain *Sargassum horneri* extract, and it was applied to *Neopyropia yezoensis* to evaluate the effect on physiological activity. Metabolites of *Sargassum horneri* were extracted by using four different methods: boiling (SBE), soaking (SSE), autoclaving (SAE) and ethanol (SEE). The SBE, SSE and SAE derived extracts showed increased tolerance to high-temperature stress that had inhibited the growth of *Neopyropia yezoensis*, and show improved growth compared to the control group. The SBE and SSE extraction methods improved the content of phycobiliprotein, but also the SBE increased superoxide dismutase (SOD) activity. Based on the results of this study, the boiling extraction method appears to be the most suitable method for the extraction of plants stimulants from *Sargassum horneri.*

## Introduction

Seaweeds have been extensively cultivated in Asian countries including China, Indonesia, Korea, and Japan^1^. The seaweed aquaculture industry is growing rapidly in Americas and Europe, with cultivation gaining interest boosted by applications for food, feed, cosmeceuticals, nutraceuticals and most recently for biofuels^2–4^. Korea is the third largest producer of seaweed in the world after China and Indonesia^1^. *Neopyropia* spp. (primarily *N. yezoensis*) (Rhodophyta) are the major aquaculture species, followed by *Undaria pinnatifida* and *Saccharina japonica* (Phaeophyceae), with over 500,000 tons of production with an economic value of $ 600 million in Korea^5^.

The rapid population expansion worldwide, together by challenges posed by extreme weather conditions have created increased challenges in food production and food security. Seaweed aquaculture has been suggested as an important source in the global food security equation^6^. However, as with agriculture, the seaweed aquaculture industry is threatened by the climate change, particularly global warming^7–9^. Among many environmental stressors, the rise in sea surface temperature is a major stressor, reducing production and quality of seaweeds. For example, the growth rate, phycoerythrin, and nitrogen removal capacity of *Neopyropia leucosticta* and *Porphyra linearis* decreases as temperature increases from 10 to 20 °C^9^. *Neopyropia tenera* grows slower and deteriorates as temperature increases from 12 to 25 °C. Chlorophyll *a* and phycocyanin of *N. tenera* gradually decreases when the species is cultured at 25 °C for prolonged periods of time^10^. High temperature also causes oxidative damage by triggering intracellular reactive oxygen species (ROS) in algae^11–14^.

Several attempts have been conducted to develop mutant cultivars of *Neopyropia* spp. that have enhanced thermal resiliency and tolerance but have not been successful. However, improvement in thermal tolerance by seaweed derived biostimulants for red algae is in the early stages of development, however these biostimulants have already been demonstrated to enhance thermal tolerance in *Ecklonia* sp. and *Saccharina* spp. (Phaeophyceae)^10,15–17^. For instance, Umanzor et al.^16^ exposed *Saccharina latissima* and *S. angustissima* to *Ascophyllum* (Phaeophyceae) (Acadian) Marine plant extract powder (AMPEP, Acadian Seaplants, LLC.), a commercially available extract. They reported that AMPEP treated sporophytes exhibited higher survival and growth at sub-optimal temperature (18 °C). The blades even became 3 times thicker in comparison to the plants without treatment. Another commercial extract, Kelpak^®^ from the brown alga, *Ecklonia maxima* also enhanced the survival of the same species at the same temperature^17^. The red alga, *Kappaphycus alvarezii* exposed to AMPEP was also improved biotic and abiotic stress tolerance, resulting in higher productivity and quality^18^. The application of seaweed derived biostimulants onto cultures of *Neopyropia* spp. while in the nursery may be an alternative to developing cultures with higher thermal tolerance that merits further exploration.

Recent and more frequent, algal blooms of *Sargassum horneri* (Phaeophyceae), also called ‘golden tides’, are responsible for negative impacts on maritime industries (aquaculture) and tourism in China and Korea^19^. What to do with all the excess biomass of this species is major problem in the region. *S. horneri* has not been studied as a source of biostimulants, but other *Sargassum* species have been suggested as a potential source for biostimulants applicable to farming^19,20^. Namely, lentils (*Lens esculenta*) exposed to *Sargassum vulgare* extract (5%), showed an improvement in growth^21^. *Sargassum angustifolium* extract improved shoot length and dry weight of rapeseed (*Brassica napus*) and also enhanced photosynthetic pigment content, free radical scavenging and superoxide dismutase (SOD) activity under desiccation stress^22^. These enhancements varied depending on the extraction method even if the same species was used^23–27^. When *Solanum lycopersicum* (tomato) was treated with extracts of *Ascophyllum nodosum*, the concentrations of trace elements (Fe, Mn, Cu and Zn) and the expression levels of growth-related hormones were significantly different. The alkaline extract of *Ascophyllum nodosum* significantly increased the dry weight of the roots. The acidic extract of *Ascophyllum nodosum* increased antioxidant enzyme activity such as CAT and SOD in roots^27^. The present study examined possible differences associated with four distinct extraction methods to obtain *Sargassum horneri* biostimulants, which were applied to the economically important red alga, *Neopyropia yezoensis*. Assessments were conducted by measuring the specific growth rate, antioxidant enzyme activities and total protein content of *N. yezoensis* treated with these extracts for 10 days.

## Method and materials

### Collection of seaweeds

The *Neopyropia yezoensis* strain (NY-HN-ST1) was originally collected from a commercial farm at Haenam, Korea (34° 57′N, 126° 60′E) in December 2020. This strain was vegetatively propagated at the Marine Ecology and Green Aquaculture Laboratory, Incheon National University using a BF-400PGC plant growth chamber (Biofree, Bucheon, Korea) at optimal growth conditions before used^28,29^. Growing conditions included von Stosch enriched medium (VSE), 10 °C, 90 ± 10 μmol m^−2^ s^−1^ photosynthetically active radiation (PAR) provided by cool white, fluorescent bulbs, 12:12 L:D, and 30 psu of salinity.

### Preparation of biostimulants

*Sargassum horneri* was collected at Jumunjin, Gangwon, Korea (37° 90′N, 128° 83′E) in June 2020. The fresh biomass was thoroughly washed with tap water to remove epiphytes, sand, and debris. Clean biomass was then dried and ground finely into powder. The first was prepared by autoclaving (SAE) according to Zahra et al.^30^ with some modifications. In short, 3 g of powder was added to 300 mL of distilled water, stirred, and autoclaved at 121 °C for 15 min. The extract was cooled at room temperature and centrifuged at 2220*g* for 10 min. The supernatant was obtained as a liquid extract. The second extract, hereafter ethanol extract (SEE), was prepared according to Motshakeri et al.^31^ with slight modification. 250 g of powder was added to 2.5 L of distilled water and shaken occasionally at room temperature for 72 h, and then filtered with Whatman no. 1 filter paper with 11 µm pore size and concentrated with a rotary vacuum evaporator (R-210, Buchi, Switzerland) at 40 °C. As a result, dried powder was obtained, which was used in experiments. Soaking and boiling extracts were prepared according to Godlewska et al.^23^ with some modification. Both extracts were prepared by adding 3 g of powder to 300 mL of distilled water. To obtain a third extract, i.e., soaking extract (SSE), 3 g of powder was soaked in 300 mL of distilled water for two days at room temperature. Lastly, a fourth extract, called boiling extract (SBE), was obtained by boiling 3 g of powder with 300 mL of distilled water for 30 min in a water bath and cooled at room temperature. Both extracts were centrifuged at 3134*g* for 25 min. The supernatant was filtered with Whatman no.1 filter paper with 11 µm pore size and the final liquid extract was then obtained.

### Experimental design

*Neopyropia yezoensis* collected in field was cultured in 2 L glass cylinder at a stocking density of 1 g L^−1^. For SBE, SEE, SSE, and SAE treatments, samples were exposed to 10% of each extract for 10 days at 10 °C, 90 ± 10 μmol m^−2^ s^−1^ photosynthetically active radiation (PAR) provided by cool white, fluorescent bulbs and 12:12 L:D. The control included only sterilized seawater and VSE medium without extract. The culture medium was renewed every 5 days to avoid nutrient limitation. The salinity was kept constant at 30 psu. After the exposure to extracts, *N. yezoensis* samples were washed in sterilized seawater to remove residues. The washed *N. yezoensis* was cultivated at two different temperatures, 10 or 20 °C, in a 500 mL Erlenmeyer flask with a stocking density of 1 g L^−1^ using VSE medium with 0.25% (w/v) germanium dioxide^29^. The samples were cultivated for 15 days, and the medium was changed every 5 days. On the same day, fresh weight of thalli was measured to calculate specific growth rate. The superoxide dismutase (SOD), catalase (CAT), ascorbate peroxidase (APX), glutathione reductase (GR), reactive oxygen species (ROS), hydrogen peroxide (H_2_O_2_), lipid peroxidation (LPO), total phenol, total protein, and pigment (Chlorophyll *a*, phycobiliprotein) were analyzed at the end of the experiment. Specific growth rates (SGR) were calculated using the following equation,

$$ SGR \;(\% /d) = \frac{{ln \;W_{end} - ln \;W_{initial} }}{{T_{end} - T_{initial} }} \times 100 $$

where W_end_ and W_initial_ represent the weights of the thalli on days T_end_ and T_initial_.

### Analysis of antioxidant enzyme activity

Fresh thalli (approximately 100 mg) were ground in 1 mL potassium phosphate buffer (50 mM, pH 7.0) containing 0.25% Triton X-100 and 1% polyvinylpyrrolidone on ice using motor driven tissue grinder. The homogenate was centrifuged at 12,000*g* for 10 min at 4 °C. The supernatant was used to measure the activity of SOD, CAT, GR, APX, total protein and ROS. Enzyme activities were measured in triplicate.

The protein content was determined based on Bradford^32^. Briefly, 25 µL enzyme extract was added with 75 µL of distilled water and 2.5 mL of Bradford’s reagent (0.025 g Coomassie Blue dye, 12.5 mL of 95% (v:v) ethanol and 25 mL H_3_PO_4_ diluted to 250 mL with distilled water). Reagents were vortexed for proper mixing and allowed for 5 min prior to taking absorbance at 595 nm. Total protein content was measured using bovine serum albumin (BSA) as standard and expressed as mg/g.

Superoxide dismutase (SOD; E.C. 1.15.1.1) activity was measured according to Misra and Fridovich^33^. Briefly, 20 µL enzyme extract was added to 150 µL carbonate buffer (pH 10.2). The production of adrenochrome due to autoxidation of epinephrine was measured at 480 nm over 3 min against a blank and activity was expressed as U/mg protein.

Catalase (CAT; E.C. 1.11.1.6) activity was measured according to Dhindsa et al.^34^. Briefly, 100 µL enzyme extract was added to 750 µL potassium phosphate buffer (50 mM, pH 7.0), 500 µL distilled water and 150 µL H_2_O_2_ (0.1 M). Absorbance was read at 240 nm at 0 and 2 min. Activity was estimated using the molar extinction coefficient of H_2_O_2_ (0.043 mM^−1^ com^−1^) and expressed as U/mg protein.

Glutathione reductase (GR; E.C. 1.8.1.7) activity was measured according to Ross and Alstyne^35^. Briefly, 15 µL enzyme extract was added with 600 µL Tris–HCl buffer (100 mM, pH 7.8) containing 100 µM NADPH, 1 mM EDTA and 0.5 mM oxidized glutathione. Absorbance was measured at 340 nm for 3 min against blank. Enzyme activity was calculated using extinction coefficient of NADPH (6.2 mM/cm) and expressed as U/mg protein.

Ascorbate peroxidase (APX; E.C. 1.11.1.11) activity was measured according to Murshed et al.^36^. Briefly, 10 µL enzyme extract was added to 185 µL reaction buffer (50 mM potassium phosphate buffer, 0.25 mM ascorbic acid). After shaking for 5 s to determine nonspecific ascorbate degradation, absorbance was measured at 290 nm at 25 °C for 3 min. Then, 5 µL H_2_O_2_ (200 mM) was added, shaken for 5 s, and then measured at 290 nm at 25 °C for 5 min. Enzyme activity was calculated using extinction coefficient of 2.8 mM^−1^ cm^−1^ and expressed as U/mg protein.

### Analysis of oxidative stress parameters

Fresh thalli (approximately 100 mg) were ground with 1 mL 10% (w:v) trichloroacetic acid (TCA) solution on ice using motor driven tissue grinder. The homogenate was centrifuged at 7000*g* for 10 min at 4 °C. The supernatant was used to determine the H_2_O_2_ and lipid peroxidation (LPO) levels. Oxidative stress parameters were measured in triplicate.

Reactive oxygen species (ROS) was measured according to Cathcart et al.^37^. Briefly, 20 µL extract was added to 180 µL sample buffer and 200 µL dye solution (1 mM DCFDA, 0.01 N NaOH and 25 mM, pH 7.2 sodium phosphate buffer). Fluorescence absorbance (λ_ex_ = 485 nm and λ_em_ = 535 nm) was measured after 1 h incubation at 20 °C in dark condition. ROS level was expressed as U/mg protein.

H_2_O_2_ was measured according to Sergiev et al.^38^. Briefly, 50 µL supernatant was added to 150 µL potassium phosphate buffer (50 mM, pH 7.0) and 100 µL potassium iodide (1 M). Absorbance was measured at 390 nm against blank. H_2_O_2_ level was quantified using H_2_O_2_ standard curve and expressed as nmol/g fresh weight.

Lipid peroxidation (LPO) level was detected by measuring the malondialdehyde (MDA) content according to Heath and Packer^39^. Briefly, 100 µL supernatant was added to 2 mL thiobarbituric acid (TCA) and then placed in a water bath for 45 min at 95 °C. Finally, the mixture was centrifuged at 4000*g* for 10 min (if necessary) and absorbance was measured at 532 nm against blank. LPO level was expressed as µmol MDA/mg protein using extinction coefficient of 156 mM^−1^ cm^−1^.

### Analysis of total phenol content and pigment

Fresh thalli (approximately 20 mg) were homogenized with 2 mL ice-cold 95% (w:w) methanol using motor driven tissue grinder. The homogenate was incubated at room temperature in the dark for 48 h and centrifuged at 13,000*g* for 5 min. The supernatant was used to determine the total phenol content and chlorophyll *a*. All measurements were performed in triplicate.

Total phenol content was measured according to Ainsworth and Gillespie^40^. Briefly, 100 µL supernatant was added to 200 µL of 10% (v:v) Folin–Ciocalteu reagent and then vortexed thoroughly. Approximately 800 µL of Na_2_CO_3_ (700 mM) was added and incubated at room temperature for 2 h, and absorbance was measured at 765 nm. The phenol content was quantified using a garlic acid standard curve and expressed mg GAE/g fresh weight.

Chlorophyll *a* was estimated based on Lichtenthaler and Wellburn^41^. Briefly, supernatant was measured at 666 and 653 nm respectively and expressed as mg/g fresh weight. Phycobiliprotein (phycoerythrin, PE and phycocyanin, PC) were extracted using 2 mL sodium phosphate buffer (50 mM, pH 6.7) based on Lin and Stekoll^42^. After homogenizing using a motor driven tissue grinder, the sample was centrifuged at 14,000*g* for 30 min at 4 °C. The supernatant was measured at 568, 620 and 730 nm, respectively and expressed as mg/g fresh weight.

### Statistical analysis

Two-way ANOVA followed by Tukey’s test (p < 0.05) was performed to examine the effect of temperature, extract, and their interaction. Normality (Sapiro–Wilk test), variable independence (Durbin–Watson test), and homogeneity of variance (Levene’s test) were checked for each factor and level. The data did not require transformation. Data are presented as mean ± standard deviation. All statistical analyses were performed using Statistical Package for the Social Science (SPSS) program version 25 (SPSS Inc., Chicago Illinois, USA).

## Results and discussion

### Effects of biostimulant and temperature on growth

**Figure 1:**
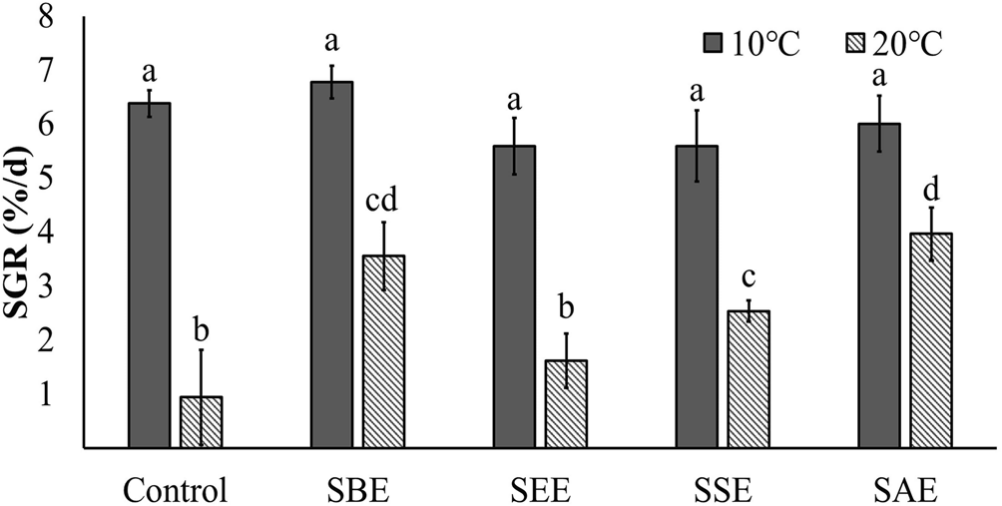
Specific growth rate (SGR) of *Neopyropia yezoensis* at different temperatures (10 and 20 °C) and four different extraction methods using *Sargassum horneri* as the source of extracts. Different letter on the bar indicates significant differences (p < 0.05). Error bars mean ± SD of triplicate. Control (without extract), *S. horneri* boiling extract (SBE), *S. horneri* ethanol extract (SEE), *S. horneri* soaking extract (SSE) and *S. horneri* autoclaving extract (SAE).

Growth rates at 10 °C were higher than those at 20 °C (Fig. 1). *Neopyropia yezoensis* showed similar growth rates at 10 °C regardless of the extraction methods. At 20 °C, however, SBE and SAE had the highest growth rates, followed by SSE, SEE and control (Fig. 1, p < 0.004). The growth rate was significantly affected by the interaction of extraction method and temperature (Table 1, p < 0.001). These results confirm that 20 °C have negative effects on the growth of *N. yezoensis* compared to 10 °C. Yamamoto et al.^43^ also found that the growth of *Neopyropia yezoensis* was lower at 20 °C than at 10 °C. Results shown here provide clear evidence that *S. horneri* extracts have a positive effect on thermal tolerance in *N. yezoensis*. Similar to our findings, studies on land plants have shown similar enhancements in tolerance to physical stress provided by extracts from other species within *Sargassum.* For example, an *Sargassum vulgare* extract increased the germination and growth of *Durum triticum*, and the seed of tomato (*Solanum lycopersicum*) under saline stress^44,45^. Also, pretreatment with *Sargassum latifolium* extract (1.5%) mitigated a damage from drought stress in *Triticum aestivum*^46^.

**Table 1:** Assessments of growth, pigments, total phenol content, total protein content, antioxidants and oxidative stress parameters of *Neopyropia yezoensis* using two-way ANOVAs as a function of temperature (10 and 20 °C), extraction methods (SBE, SWE, SSE and SAE), and their interaction. Significant differences are bolded.

Parameter	Temperature			Extraction method			Temperature * Extract method		
	df	*F*	*p*	df	*F*	*p*	df	*F*	*p*
SGR	1	248.974	**< 0.001**	4	9.929	**< 0.001**	4	7.622	**< 0.001**
Chlorophyll *a*	1	153.391	**< 0.001**	4	3.779	**< 0.05**	4	5.325	**< 0.05**
Phycocyanin	1	48.891	**< 0.001**	4	7.961	**< 0.001**	4	5.809	**< 0.05**
Phycoerythrin	1	61.736	**< 0.001**	4	13.796	**< 0.001**	4	7.365	**< 0.001**
Phenol	1	9.765	**< 0.05**	4	2.150	> 0.05	4	0.641	> 0.05
Protein	1	8.436	**< 0.05**	4	1.195	> 0.05	4	1.681	> 0.05
SOD	1	31.492	**< 0.001**	4	19.434	**< 0.001**	4	12.138	**< 0.001**
CAT	1	49.865	**< 0.001**	4	2.359	> 0.05	4	1.312	> 0.05
GR	1	176.683	**< 0.001**	4	4.529	**< 0.05**	4	3.902	**< 0.05**
APX	1	53.845	**< 0.001**	4	9.781	**< 0.001**	4	2.179	> 0.05
ROS	1	6.859	**< 0.05**	4	8.158	**< 0.001**	4	3.886	**< 0.05**
H_2_O_2_	1	9.358	**< 0.05**	4	3.741	**< 0.05**	4	3.565	**< 0.05**
LPO	1	28.696	**< 0.001**	4	0.706	> 0.05	4	2.06	> 0.05

Our study also showed that the extraction methods play a key role on the enhancements that could be provided. SBE and SAE were the most efficient method to improve the thermal tolerance in *Neopyropia yezoensis*. In terms of production of *N. yezoensis*, the daily production in the control and SBE at 10 °C was 0.108 and 0.118 g L^−1^, respectively. The daily production values at 20 °C were reduced to 0.015 and 0.043 g L^−1^, respectively. In other words, SBE developed in this study can increase the production of *N. yezoensis* by 185%, compared to the control at 20 °C. The boiling method (SBE) has been considered a good method of extraction in other studies^23^. Godlewska et al.^23^ compared extractions using *Polysiphonia* (Rhodophyta), *Ulva* and *Cladophora* (Chlorophyta) by boiling and soaking methods. Ten percent boiling extract showed the highest growth results for garden cress (*Lepidium sativum*). The extract (50%) produced by boiling *Ulva lactuca* (formerly *Ulva fasciata*)*, Sargassum ilicifolium* (Phaeophyceae) and *Gracilaria corticata* (Rhodophyta) in distilled water had a positive effect on fresh weight and total nitrogen when treated with *Trigonella foenum-graecum* than the extract (50%) produced by soaking^47^. The boiling extract contains important inorganic nutrients (N, P, S and B) than the soaking extract and is reported to contain higher polyphenols^24^. In addition, the boiling and autoclaving extracts of brown algae, *Sargassum* sp. contain higher polyphenols compared to green and red algae^48^. Polyphenols are known to be effective antioxidants^49^. Organic compounds such as organic acid, methionine, polyamines, polyphenol and mannitol contained in brown algae chelate available nutrients, increasing nutrient absorption and allowing effective nutrient use^49,50^.

### Effects of biostimulant and temperature on pigments

**Figure 2:**
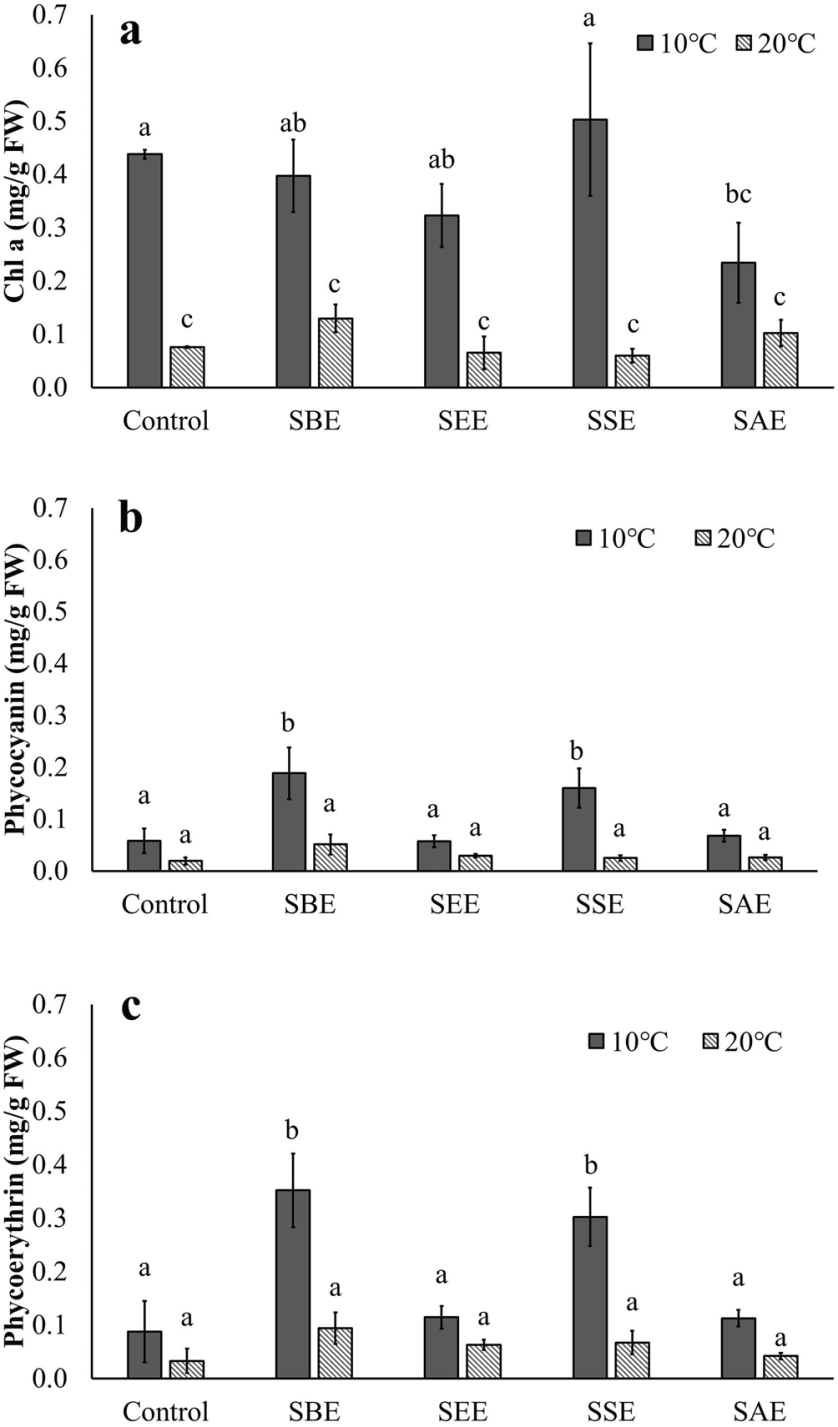
Chlorophyll *a* (**a**), phycocyanin (**b**) and phycoerythrin (**c**) of *Neopyropia yezoensis* at different temperatures (10 and 20 °C) and four different extraction methods using *Sargassum horneri* as the source of extract. Different letter on the bar indicates significant differences (p < 0.05). Error bars mean ± SD of triplicate. Control (without extract), *S. horneri* boiling extract (SBE), *S. horneri* ethanol extract (SEE), *S. horneri* soaking extract (SSE) and *S. horneri* autoclaving extract (SAE).

Chlorophyll *a* assays showed similar results for SBE, SEE, SSE, and the controls at 10 °C, with the only significant difference detected in SAE (Fig. 2a). Chlorophyll *a* contents were significantly lower at 20 °C than at 10 °C and no positive effects of *Sargassum* extracts were observed (Fig. 2a, p > 0.05). According to the results of present study, the content of chlorophyll *a* appears to decrease at high temperatures. Similar to these results, it was reported that the high temperature of 32–36 °C significantly reduced the photosynthetic pigment in the red alga, *Kappaphycus alvarezii*^51^. Phycocyanin and phycoerythrin contents were significantly higher at SBE and SSE at 10 °C than all other conditions (p < 0.001) and no enhancement in thermal tolerance by the extracts were observed (Fig. 2b,c, p > 0.05). Chlorophyll *a* and phycobiliprotein were significantly affected by the interaction of temperature and the extract (Table 1, p < 0.05). According to a previous study, the concentration of phycobiliprotein in *Crassiphycus caudatus* (formerly *Gracilaria caudata*) (Rhodophyta) treated with 5 g L^−1^ of AMPEP was stimulated. The application of seaweed derived biostimulants may increase the concentration of phycobiliprotein in red algae^52^.

### Effects of biostimulant and temperature on biochemical responses

**Figure 3:**
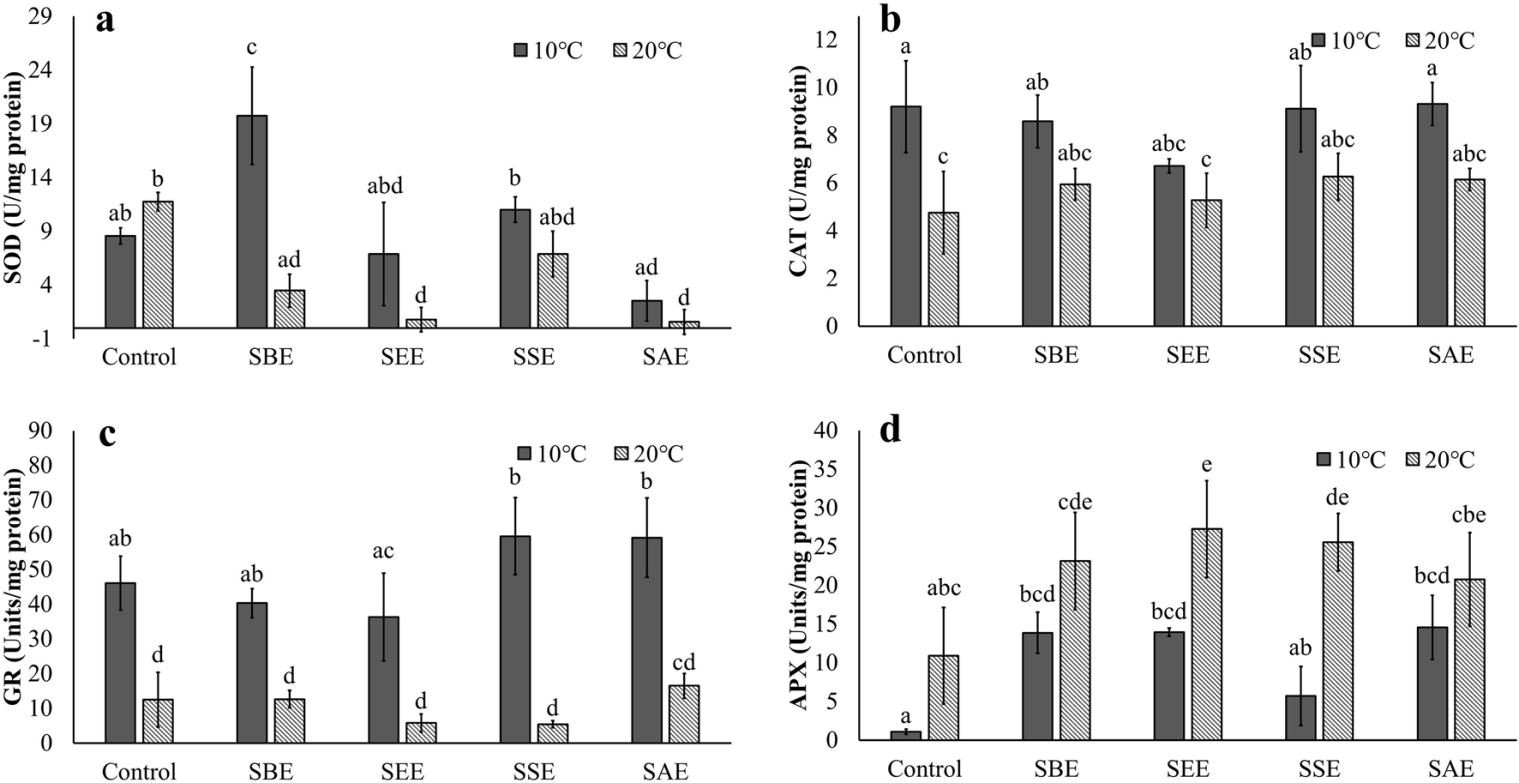
Superoxide dismutase (SOD; **a**), catalase (CAT; **b**), glutathione reductase (GR; **c**), ascorbate peroxidase (APX; **d**) of *Neopyropia yezoensis* at different temperatures (10 and 20 °C) and four different extraction methods using *Sargassum horneri* as source of extract. Different letter on the bar indicates significant differences (p < 0.05). Error bars mean ± SD of triplicate. Control (without extract), *S. horneri* boiling extract (SBE), *S. horneri* ethanol extract (SEE), *S. horneri* soaking extract (SSE) and *S. horneri* autoclaving extract (SAE).

To evaluate oxidative stress, enzymatic (SOD, CAT, GR, and APX) and non-enzymatic (total phenol) antioxidant activity were measured. At 10 °C, SOD activity was highest in SBE, and all other extracts were similar to control. At 20 °C, the SOD in SSE was similar to the control and all other extraction methods showed lower SOD than the control (Fig. 3a). Temperature stress induces oxidative stress. SOD is the first enzyme affecting the decryption pathway for ROS^53^. Enhancement of SOD activity of *Neopyropia yezoensis* by SBE in *Sargassum horneri* extract was observed (Fig. 3a). Treatment with biostimulants derived from brown seaweeds appears to be able to increase SOD activity in land crops. For example, *Ascophyllum nodosum* extract stimulated SOD activity under drought stress of the plant, *Paspalum vaginatum*^54^. Another *Ascophyllum nodosum* based product, Tasco-Forage, also increased the SOD activity in Kentucky bluegrass (*Poa pratensis*)^55^. As shown in previous work, the SOD activity of *Ulva australis* and *Neopyropia yezoensis* decreases over time when these species are subject to salinity stress^56^. *Neopyropia yezoensis* in the present study also decreased the SOD activity when high temperature treatments. SBE, however, increased their SOD activity at the optimal temperature of 10 °C. It has been reported through several studies that treatment with biostimulant increases the activity of SOD in the absence of stress^54,57^. SOD is a metalloprotein and includes three isoforms determined by metal-center cofactors: Cu/Zn SOD, Mn SOD and Fe SOD^12,58^. In previous studies, it was reported that different extraction methods contained different trace elements^23,27^. As previously mentioned, SBE is rich in organic compounds (i.e., polyphenol, organic acid) for chelating, with can increase the uptake of trace metals for SOD synthesis (Supplementary Information).

Furthermore, CAT is directly involved in photosynthetic reactions^59^. It is a temperature-sensitive enzyme, and its activity decreases with increasing temperature^60^. The present study also showed a significant effect of temperature in the CAT activity (Fig. 3b, p < 0.001). However, the extraction method did not have a significant effect and no interaction between temperature and the extraction method was detected (Fig. 3b and Table 1, p > 0.05).

GR is an enzyme in the Ascorbate–Glutathione (AsA-GSH) cycle and functions as a necessary function in the defense system against ROS by maintaining the reduced state of glutathione. A decrease in GR activity was recorded with increasing temperature in *Synechocystis* PCC-6803^61^. GR activity was significantly lower at 20 °C compared to 10 °C and regardless of the extract (Fig. 3c, p < 0.045). A decrease in GR activity with increasing temperature was observed in the mung bean, *Vigna radiata*^62^. According to Schmidt and Kunert^63^, GR in *Phaseolus vulgaris* (kidney beans) increased vitamin C and glutathione as a primary reaction after increased lipid peroxidation, followed by increased activity. A higher increase in lipid peroxidation at 10 °C than at 20 °C in *Neopyropia yezoensis* in our study may support the increased activity of GR at the temperature of 10 °C.

**Figure 4:**
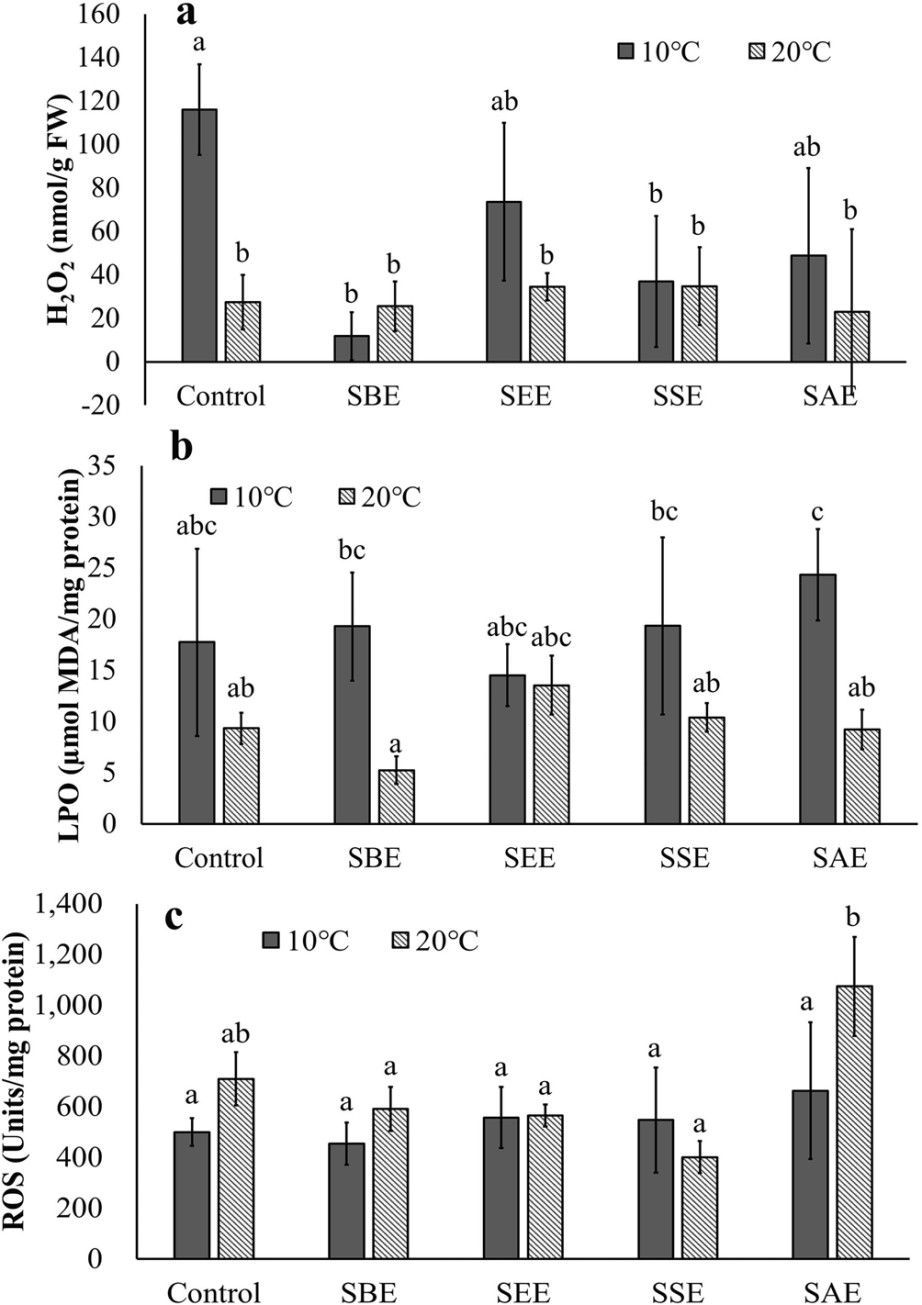
Hydrogen peroxide (H_2_O_2_; **a**), lipid peroxidation (LPO; **b**) and reactive oxygen species (ROS; **c**) of *Neopyropia yezoensis* at different temperatures (10 and 20 °C) and four different extraction methods using *Sargassum horneri.* Different letter on the bar indicates significant differences (p < 0.05). Error bars mean ± SD of triplicate. Control (without extract), *S. horneri* boiling extract (SBE), *S. horneri* ethanol extract (SEE), *S. horneri* soaking extract (SSE), and *S. horneri* autoclaving extract (SAE).

On the other hand, APX activity showed an opposite trend to GR activity (Fig. 3d). The APX activity was higher at 20 °C compared to 10 °C. All *Sargassum* extracts showed some enhancement of APX activity in comparison to the control at both temperatures (p < 0.036). A higher plant, *Arabidopsis thaliana* also showed some increase of APX activity when experienced temperature stress (e.g., 22 °C)^64^. Other plants, including *Carrizo citrange* and *Cleopatra mandarin* showed higher sensitivity to heat stress than to drought stress in terms of APX activity^65^. APX is an important component of the AsA-GSH cycle and catalyzes the conversion of H_2_O_2_ to H_2_O^53^. The reduction of H_2_O_2_ at 20 °C in the present study may be due to the higher activity of APX (Figs. 3d and 4a). APX has also been reported to help reduce the rate of lipid peroxidation in *Arabidopsis thaliana*^66^. Experiments conducted on *Carapa guianensis* indicate that the APX and CAT activity attenuated stress-induced lipid peroxidation through a positive regulation^67^. These results explain the lower LPO at 20 °C in the present study (Fig. 4b).

**Figure 5:**
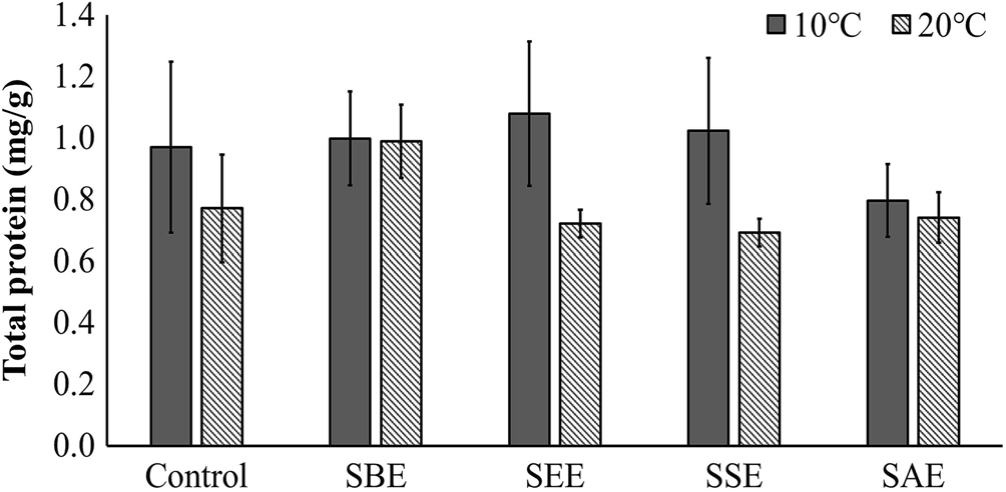
Total protein of *Neopyropia yezoensis* at different temperatures (10 and 20 °C) and four different extraction methods using *Sargassum horneri* as extract source. Different letter on the bar indicates significant differences (p < 0.05). Error bars mean ± SD of triplicate. Control (without extract), *S. horneri* boiling extract (SBE), *S. horneri* ethanol extract (SEE), *S. horneri* soaking extract (SSE) and *S. horneri* autoclaving extract (SAE).

**Figure 6:**
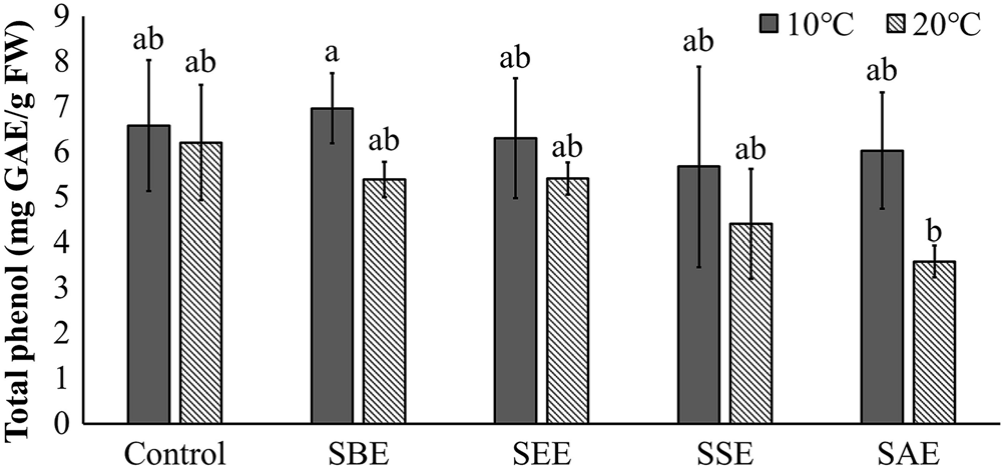
Total phenol of *Neopyropia yezoensis* at different temperatures (10 and 20 °C) and four different extraction methods using *Sargassum horneri* as the source of extract. Different letter on the bar indicates significant differences (p < 0.05). Error bars mean ± SD of triplicate. Control (without extract), *S. horneri* boiling extract (SBE), *S. horneri* ethanol extract (SEE), *S. horneri* soaking extract (SSE) and *S. horneri* autoclaving extract (SAE).

The ROS values obtained here, did not show significantly differences as a function of temperature (Fig. 4c, p > 0.05), suggesting that antioxidant enzymes relieved oxidative stress effectively and regulated the ROS balance. A balanced ROS also serves as an important signal modulator in stress conditions rather than a lethal role^68–70^. Total protein content and total phenol content were affected only by temperature, and there was no significant effect by the extraction method or the interaction between temperature and extract (Figs. 5 and 6, Table 1, p > 0.05).

## Conclusion

This study confirms that *Sargassum* extracts, particularly of *S. horneri* can be a new source of biostimulants that enhance the thermal tolerance of *Neopyropia yezoensis*. This study also confirms that the extraction method to obtain biostimulants plays a key role for the effectiveness of the extract. Among the extraction methods, boiling (SBE) and soaking (SSE) of finely ground tissue are the most effective methods to provide growth and phycobiliproteins content enhancements in *N. yezoensis*. Regardless of the extraction method used, the expression levels of antioxidant enzymes and oxidative stress were dominated by temperature. A quantitative analysis of molecules/chemical compounds from different extraction methods should be conducted to determine what molecules/chemical compounds in the extracts stimulate the growth and temperature stress tolerance. It is also important to note that the chemical composition of *Sargassum horneri* may exist in different populations. However, given the positive results obtained here, it would be recommended that *Sargassum horneri* extracts be tested on other economically important seaweeds, such as *Undaria pinnatifida* and *Saccharina japonica.*

## Supplementary Information

Supplementary Information.

## References

[1] Kim JK, Yarish C, Hwang EK, Park M, Kim Y (2017). Seaweed aquaculture: Cultivation technologies, challenges and its ecosystem services. Algae.

[2] Park JS (2021). Evaluation of nutrient bioextraction by seaweed and shellfish aquaculture in Korea. J. World Aquacult. Soc..

[3] Kim JK, Stekoll M, Yarish C (2019). Opportunities, challenges and future directions of open-water seaweed aquaculture in the United States. Phycologia.

[4] FAO. *The State of World Fisheries and Aquaculture 2020* (2020).

[5] FAO. Fisheries and aquaculture statistics. https://www.fao.org/fishery/statistics-query/en/capture/capture_quantity (2021).

[6] Yarish, C. *et al.* Seaweed aquaculture for food security, income generation and environmental health in tropical developing countries. *Banq. Mond.* (2016).

[7] Tang Q, Zhang J, Fang J (2011). Shellfish and seaweed mariculture increase atmospheric CO_2_ absorption by coastal ecosystems. Mar. Ecol. Prog. Ser..

[8] Duarte CM, Wu J, Xiao X, Bruhn A, Krause-Jensen D (2017). Can seaweed farming play a role in climate change mitigation and adaptation?. Front. Mar. Sci..

[9] Kim JK, Kraemer GP, Neefus CD, Chung IK, Yarish C (2007). Effects of temperature and ammonium on growth, pigment production and nitrogen uptake by four species of *Porphyra* (Bangiales, Rhodophyta) native to the New England coast. J. Appl. Phycol..

[10] Lee H-J, Choi J-I (2019). Enhancing temperature tolerance of *Pyropia tenera* (Bangiales) by inducing mutation. Phycologia.

[11] Lesser MP (2006). Oxidative stress in marine environments: Biochemistry and physiological ecology. Annu. Rev. Physiol..

[12] Mallick N, Mohn FH (2000). Reactive oxygen species: Response of algal cells. J. Plant Physiol..

[13] Dummermuth A, Karsten U, Fisch K, König G, Wiencke C (2003). Responses of marine macroalgae to hydrogen-peroxide stress. J. Exp. Mar. Biol. Ecol..

[14] Bischof K, Rautenberger R, Wiencke C, Bischof K (2012). Seaweed responses to environmental stress: Reactive oxygen and antioxidative strategies. Seaweed Biology: Ecological Studies.

[15] Ding H, Zhang B, Yan X (2016). Isolation and characterization of a heat-resistant strain with high yield of *Pyropia yezoensis* Ueda (Bangiales, Rhodophyta). Aquac. Fish..

[16] Umanzor S, Shin S, Yarish C, Augyte S, Kim JK (2020). Exploratory evaluation of the effects of Kelpak^®^ seaweed extract on cultivated kelp *Saccharina* spp. exposed to sublethal and lethal temperatures. J. World Aquacult. Soc..

[17] Umanzor S (2019). Preliminary assessment on the effects of the commercial seaweed extract, AMPEP, on growth and thermal tolerance of the kelp *Saccharina* spp. from the Northwest Atlantic. J. Appl. Phycol..

[18] Hurtado AQ, Critchley AT (2018). A review of multiple biostimulant and bioeffector benefits of AMPEP, an extract of the brown alga *Ascophyllum nodosum*, as applied to the enhanced cultivation and micropropagation of the commercially important red algal carrageenophyte *Kappaphycus alvarezii* and its selected cultivars. J. Appl. Phycol..

[19] Byeon SY (2019). The origin and population genetic structure of the ‘golden tide’ seaweeds, *Sargassum horneri*, in Korean waters. Sci. Rep..

[20] Byeon SY (2020). Comparative analysis of sequence polymorphism in complete organelle genomes of the ‘Golden Tide’ seaweed *Sargassum horneri* between Korean and Chinese forms. Sustainability.

[21] Mendoza-Morales LT, Mendoza-González AC, Mateo-Cid LE, Rodríguez-Dorantes A (2019). Analysis of the effect as biostimulants of *Sargassum vulgare* and *Ulva fasciata* extracts on *Lens esculenta* growth. Mex. J. Biotechnol..

[22] Shahriari AG, Mohkami A, Niazi A, Parizipour MHG, Habibi-Pirkoohi M (2021). Application of brown algae (*Sargassum angustifolium*) extract for improvement of drought tolerance in Canola (*Brassica*
*napus* L.). Iran. J. Biotechnol..

[23] Godlewska, K., Michalak, I., Tuhy, Ł. & Chojnacka, K. Plant growth biostimulants based on different methods of seaweed extraction with water. *Biomed Res. Int.* **2016** (2016). 10.1155/2016/5973760 PMC4913069 27366749

[24] Michalak I, Tuhy Ł, Chojnacka K (2015). Seaweed extract by microwave assisted extraction as plant growth biostimulant. Open Chem..

[25] Tierney MS (2013). Influence of pressurised liquid extraction and solid–liquid extraction methods on the phenolic content and antioxidant activities of Irish macroalgae. Int. J. Food Sci. Technol..

[26] Prasedya, E. S. *et al.* Effect of solid and liquid extract of *Sargassum crassifolium* on growth and yield of rice plant. in *AIP Conference Proceedings* **2199**, 070007 (AIP Publishing LLC, 2019).

[27] Carrasco-Gil S, Hernandez-Apaolaza L, Lucena JJ (2018). Effect of several commercial seaweed extracts in the mitigation of iron chlorosis of tomato plants (*Solanum*
*lycopersicum* L.). Plant Growth Regul..

[28] Le B (2019). Effect of silicon in *Pyropia yezoensis* under temperature and irradiance stresses through antioxidant gene expression. J. Appl. Phycol..

[29] Redmond S, Green L, Yarish C, Kim J, Neefus C (2014). New England Seaweed Culture Handbook.

[30] Zahra R, Mehrnaz M, Farzaneh V, Kohzad S (2007). Antioxidant activity of extract from a brown alga, *Sargassum*
*boveanum*. Afr. J. Biotechnol..

[31] Motshakeri, M. *et al.* Effects of brown seaweed (*Sargassum polycystum*) extracts on kidney, liver, and pancreas of type 2 diabetic rat model. *Evid. Based Complement Altern. Med.* **2014** (2014). 10.1155/2014/379407 PMC3910465 24516503

[32] Bradford MM (1976). A rapid and sensitive method for the quantitation of microgram quantities of protein utilizing the principle of protein-dye binding. Anal. Biochem..

[33] Misra HP, Fridovich I (1972). The role of superoxide anion in the autoxidation of epinephrine and a simple assay for superoxide dismutase. J. Biol. Chem..

[34] Dhindsa RS, Plumb-Dhindsa P, Thorpe TA (1981). Leaf senescence: Correlated with increased levels of membrane permeability and lipid peroxidation, and decreased levels of superoxide dismutase and catalase. J. Exp. Bot..

[35] Ross C, Alstyne KLV (2007). Intraspecific variation in stress-induced hydrogen peroxide scavenging by the ulvoid macroalga *Ulva lactuca*. J. Phycol..

[36] Murshed R, Lopez-Lauri F, Sallanon H (2008). Microplate quantification of enzymes of the plant ascorbate–glutathione cycle. Anal. Biochem..

[37] Cathcart R, Schwiers E, Ames BN (1983). Detection of picomole levels of hydroperoxides using a fluorescent dichlorofluorescein assay. Anal. Biochem..

[38] Sergiev I, Alexieva V, Karanov E (1997). Effect of spermine, atrazine and combination between them on some endogenous protective systems and stress markers in plants. Compt. Rend. Acad. Bulg. Sci..

[39] Heath RL, Packer L (1968). Photoperoxidation in isolated chloroplasts: I. Kinetics and stoichiometry of fatty acid peroxidation. Arch. Biochem. Biophys..

[40] Ainsworth EA, Gillespie KM (2007). Estimation of total phenolic content and other oxidation substrates in plant tissues using Folin–Ciocalteu reagent. Nat. Protoc..

[41] Lichtenthaler HK, Wellburn AR (1983). Determinations of total carotenoids and chlorophylls *a* and *b* of leaf extracts in different solvents. Biochem. Soc. Trans..

[42] Lin R, Stekoll MS (2011). Phycobilin content of the conchocelis phase of Alaskan *Porphyra* (Bangiales, Rhodophyta) species: Responses to environmental variables. J. Phycol..

[43] Yamamoto M, Watanabe Y, Kinoshita H (1991). Effects of water temperature on the growth of red alga *Porphyra yezoensis* form *narawaensis* (nori) cultivated in an outdoor raceway tank. Nippon Suisan Gakkaishi.

[44] Latique S, Elouaer MA, Chernane H, Hannachi C, Elkaoua M (2014). Effect of seaweed liquid extract of *Sargassum vulgare* on growth of durum wheat seedlings (*Triticum durum* L) under salt stress. Int. J. Innov. Appl. Stud..

[45] Aymen, E. M., Salma, L., Halima, C., Cherif, H. & Mimoun, E. Effect of seaweed extract of *Sargassum vulgare* on germination behavior of two tomatoes cultivars (*Solanum lycopersicum* L) under salt stress. *Octa J. Environ. Res.* **2** (2014).

[46] Kasim WA, Hamada EA, El-Din NS, Eskander S (2015). Influence of seaweed extracts on the growth, some metabolic activities and yield of wheat grown under drought stress. Int. J. Agron. Agric. Res.

[47] Pise NM, Sabale A (2010). Effect of seaweed concentrates on the growth and biochemical constituents of *Trigonella foenum-graecum* L. J. Phytol..

[48] Yangthong M, Hutadilok-Towatana N, Phromkunthong W (2009). Antioxidant activities of four edible seaweeds from the southern coast of Thailand. Plant Food Hum. Nutr..

[49] Battacharyya D, Babgohari MZ, Rathor P, Prithiviraj B (2015). Seaweed extracts as biostimulants in horticulture. Sci. Hortic..

[50] Chatzissavvidis C, Therios I, Pomin VH (2014). Role of algae in agriculture. Seaweeds: Agricultural Uses, Biological and Antioxidant Agents.

[51] Kumar YN (2020). Impact of elevated temperature on the physiological and biochemical responses of *Kappaphycus alvarezii* (Rhodophyta). PLoS One.

[52] Souza JM, Castro JZ, Critchley AT, Yokoya NS (2019). Physiological responses of the red algae *Gracilaria caudata* (Gracilariales) and *Laurencia catarinensis* (Ceramiales) following treatment with a commercial extract of the brown alga *Ascophyllum nodosum* (AMPEP). J. Appl. Phycol..

[53] Gill SS (2015). Superoxide dismutase—mentor of abiotic stress tolerance in crop plants. Environ. Sci. Pollut. Res..

[54] O Elansary H, Mahmoud EA, El-Ansary DO, Mattar MA (2020). Effects of water stress and modern biostimulants on growth and quality characteristics of mint. Agronomy.

[55] Allen V (2001). Tasco: Influence of a brown seaweed on antioxidants in forages and livestock—A review. J. Anim. Sci..

[56] Samanta P, Shin S, Jang S, Kim JK (2019). Comparative assessment of salinity tolerance based on physiological and biochemical performances in *Ulva australis* and *Pyropia yezoensis*. Algal Res..

[57] Latef AAHA, Srivastava AK, Saber H, Alwaleed EA, Tran L-SP (2017). *Sargassum muticum* and *Jania rubens* regulate amino acid metabolism to improve growth and alleviate salinity in chickpea. Sci. Rep..

[58] Rezayian M, Niknam V, Ebrahimzadeh H (2019). Oxidative damage and antioxidative system in algae. Toxicol. Rep..

[59] Eyster HC (1950). Effect of temperature on catalase activity. Ohio J. Sci..

[60] Morgulis S, Beber M, Rabkin I (1926). Studies on the effect of temperature on the catalase reaction. J. Biol. Chem..

[61] Rady AA, El-Sheekh MM, Matkovics B (1994). Temperature shift-induced changes in the antioxidant enzyme system of Cyanobacterium *Synechocystis* PCC 6803. Int. J. Biochem. Cell Biol..

[62] Nahar K, Hasanuzzaman M, Alam MM, Fujita M (2015). Exogenous glutathione confers high temperature stress tolerance in mung bean (*Vigna*
*radiata* L.) by modulating antioxidant defense and methylglyoxal detoxification system. Environ. Exp. Bot..

[63] Schmidt A, Kunert KJ (1986). Lipid peroxidation in higher plants: The role of glutathione reductase. Plant Physiol..

[64] Lokhande SD, Ogawa KI, Tanaka A, Hara T (2003). Effect of temperature on ascorbate peroxidase activity and flowering of *Arabidopsis thaliana* ecotypes under different light conditions. J. Plant Physiol..

[65] Balfagón D, Zandalinas SI, Baliño P, Muriach M, Gómez-Cadenas A (2018). Involvement of ascorbate peroxidase and heat shock proteins on citrus tolerance to combined conditions of drought and high temperatures. Plant Physiol. Biochem..

[66] Rodríguez-Martínez RE (2020). Element concentrations in pelagic *Sargassum* along the Mexican Caribbean coast in 2018–2019. Peer J.

[67] Cruz F, Castro G, Júnior DS, Festucci-Buselli R, Pinheiro H (2013). Exogenous glycine betaine modulates ascorbate peroxidase and catalase activities and prevent lipid peroxidation in mild water-stressed *Carapa guianensis* plants. Photosynthetica.

[68] Khedia J (2020). *Sargassum* seaweed extract enhances *Macrophomina phaseolina* resistance in tomato by regulating phytohormones and antioxidative activity. J. Appl. Phycol..

[69] Mittler R (2011). ROS signaling: The new wave?. Trends Plant Sci..

[70] Lehmann S, Serrano M, L’Haridon F, Tjamos SE, Metraux J-P (2015). Reactive oxygen species and plant resistance to fungal pathogens. Phytochemistry.

